# The Early Recognition of Measles

**Published:** 1910-12-10

**Authors:** 


### Diseases of Children.

### THE EARLY RECOGNITION OF MEASLES.

The fact that measles is especially infectious before the appearance of the rash renders it necessary to give careful consideration to any signs or symptoms from which the probable onset of the disease may be suspected.

After having definitely isolated a patient suffering from measles, the picking out of early cases is sometimes complicated by the presence of children, who are so often the subjects of slight symptoms, that it is difficult to know how much importance to attach to a very mild sore throat or a slight cold, or even to the spots and pimples of a child with a normally coarse and unhealthy skin.

In dealing with such cases, if the child has not previously had measles, it is wise to consider and treat it as infected; in those who have been previously attacked, some hesitation and delay are permissible, especially if the means of isolation are already being severely taxed. But in this connection it has been pointed out that an abrupt disappearance of catarrhal symptoms is suspicious; a short rise of temperature with, for example, a coryza lasting only for about twenty-four hours is not uncommonly met with during the incubation period of measles. After a few days of apparent health the unequivocal signs of measles make their appearance, the child having in the meantime been allowed to mix with others.

### Conjunctivitis.

Another well-recognised sign is the injection of the conjunctiva. It has been said that this is most often more marked on the bulbar portion, when the catarrh first begins to show, and that it radiates outwards from the edge of the cornea; but this must be of doubtful value in the differential diagnosis from other forms of conjunctivitis.

### Koplik's Spots.

Concerning the spots to be observed in the mouth, which are associated with the name of Koplik, it may be said that they are now regarded as important items in the early diagnosis of measles. These spots should therefore be looked for, and always in clear daylight, as they are easily missed in artificial light. Once seen a Koplik spot will easily be recognised again, but it is perhaps at first difficult to distinguish these spots from other small points on the mucous membrane.

The typical Koplik spot may be described as a minute greyish-white speck, surrounded by a bluish-red areola; they are most commonly found on the cheek opposite the lower molars. They appear about two days before the rash, rarely three days before, and are sometimes only to be seen one day before the exanthem.

Besides these spots, there are to be seen very commonly indeed much more easily recognised dark red patches inside the mouth; these are irregular in shape and most common on the palate, and nearly always before the rash is out, while they may be found two days before.

Among other signs in the mouth may be mentioned a slight redness or catarrh of the fauces, and a gingivitis which has been described as sometimes met with at an early date. The sore throat may be accompanied by some temporary fever, as referred to above, and perhaps a cough, possibly due to involvement of the larynx, often disappearing when the rash comes out.

### Loss of Weight.

From observations in institutions, it appears that it is sometimes possible to foretell the onset of measles by a loss of weight during the incubation period, and while the child is still in apparent health. Where it is customary to record frequently the weights of children living under routine conditions this may be of value, but a record of the variations of each child's weight over a preceding lengthy period must be available.

### Leucocytosis.

The occurrence of a leucocytosis is a point of, perhaps, less practical importance to the general practitioner; but inasmuch as it appears to be capable of demonstration some days before any signs or symptoms, the examination of the blood of suspected children might be a very useful help. The leucocytosis is of the polymorphonuclear variety, starts towards the end of the incubation period, and lasts through the period of invasion; thereafter the number falls until, during the eruptive period, the leucocytes may be normal or even less than normal in number. The most useful time to commence examining the blood would be about a week after exposure, allowing nine or ten days for the incubation period. The remission of the leucocytosis in measles is in direct contrast to the hyperleucocytosis, which is common, if not constant, in the eruptive stage of scarlet fever; but, unfortunately, the examination of the blood is full of pitfalls, especially in children, so that a good deal of caution is advisable in drawing inferences in doubtful cases.

### Further Clues.

Prodromal rashes are not rare either in adults or children, the commonest variety being a rash resembling measles itself, and leading one to think of revising dates. Another fairly common one is a punctate erythema, resembling scarlet fever, and causing some temporary hesitation in diagnosis; but these prodromal rashes are not usually well developed or very clear, and their disappearance before the true rash comes out gives them a value in diagnosis.

Among other suspicious signs, besides those commonly recognised, may be mentioned photophobia, noticeable even before there is any suffusion of the eyes or lachrymation; drowsiness, in a usually alert child, without apparent cause; and sneezing, which should be regarded as a very suggestive symptom, calling for immediate isolation. As it often occurs quite early it is a valuable warning.

